# Corneal Tissue Engineering: From Bench to Clinic

**DOI:** 10.1155/2018/6091389

**Published:** 2018-12-23

**Authors:** Sang Beom Han, Jodhbir S. Mehta, Yu-Chi Liu, Karim Mohamed-Noriega

**Affiliations:** ^1^Department of Ophthalmology, Kangwon National University Hospital, Kangwon National University School of Medicine, Chuncheon, Republic of Korea; ^2^Singapore National Eye Centre, Singapore; ^3^Singapore Eye Research Institute, Singapore; ^4^Department of Ophthalmology, Yong Loo Lin School of Medicine, National University of Singapore, Singapore; ^5^Department of Ophthalmology, Autonomous University of Nuevo Leon, Monterrey, Mexico

Corneal blindness is one of the major causes of blindness worldwide, for which corneal transplantation has long been the only treatment method. In addition to penetrating keratoplasty, development of partial thickness corneal transplantation techniques including deep anterior lamellar keratoplasty (DALK), Descemet's stripping endothelial keratoplasty (DSEK), and Descemet's membrane endothelial keratoplasty (DMEK) allowed improved visual anatomical outcome. Keratoprosthesis including Boston keratoprosthesis (KPro) and osteo-odonto keratoprosthesis (OOKP) also enabled visual recovery in patients with serious ocular surface destruction. However, the demands for corneal transplant can never be met because of global shortage of donor corneal tissue.

We have introduced in the call for papers for this special issue that various methods of corneal tissue engineering such as cultivation and expansion of corneal tissues using novel drugs and biomaterials have been studied. These novel methods are expected to allow the production of large amount of synthetic corneal tissues for transplantation without the risk of disease transmission. Further researches on corneal tissue engineering, i.e., cell therapy for corneal tissues including the epithelium, stroma, and endothelium; development of biomaterials, drugs, and imaging techniques that can be applied to corneal tissue engineering; application of artificial corneal tissue; and anterior segment reconstruction would provide even more improved visual and anatomical results.

In this special issue, the authors contributed 4 original articles and 2 review papers regarding researches of corneal tissue engineering and its clinical application.

The authors reported the results of their original researches on various topics in corneal tissue engineering: (1) outcomes of Descemet membrane endothelial keratoplasty for vitrectomized eyes with sutured posterior chamber intraocular lens; (2) generation of femtosecond laser-cut decellularized corneal lenticule using hypotonic trypsin-EDTA solution for corneal tissue engineering; (3) modern corneal eye-banking using a software-based IT management solution; and (4) preliminary investigation of the mechanical anisotropy of the normal human corneal stroma.

This special issue also includes review articles on the following topics: (1) application of novel drugs for corneal cell regeneration and (2) from DMEK to corneal endothelial cell therapy: technical and biological aspects.

We believe these articles will provide readers with valuable information on corneal tissue engineering and novel ideas for researches on related topics.

